# High transition frequencies of dynamic functional connectivity states in the creative brain

**DOI:** 10.1038/srep46072

**Published:** 2017-04-06

**Authors:** Junchao Li, Delong Zhang, Aiying Liang, Bishan Liang, Zengjian Wang, Yuxuan Cai, Mengxia Gao, Zhenni Gao, Song Chang, Bingqing Jiao, Ruiwang Huang, Ming Liu

**Affiliations:** 1 Center for the Study of Applied Psychology, Key Laboratory of Mental Health and Cognitive Science of Guangdong Province, School of Psychology, South China Normal University, Guangzhou, China; 2 Guangdong Science Center, Guangzhou, China; 3 College of Education, Guangdong Polytechnic Normal University, Guangzhou, China.

## Abstract

Creativity is thought to require the flexible reconfiguration of multiple brain regions that interact in transient and complex communication patterns. In contrast to prior emphases on searching for specific regions or networks associated with creative performance, we focused on exploring the association between the reconfiguration of dynamic functional connectivity states and creative ability. We hypothesized that a high frequency of dynamic functional connectivity state transitions will be associated with creative ability. To test this hypothesis, we recruited a high-creative group (HCG) and a low-creative group (LCG) of participants and collected resting-state fMRI (R-fMRI) data and Torrance Tests of Creative Thinking (TTCT) scores from each participant. By combining an independent component analysis with a dynamic network analysis approach, we discovered the HCG had more frequent transitions between dynamic functional connectivity (dFC) states than the LCG. Moreover, a confirmatory analysis using multiplication of temporal derivatives also indicated that there were more frequent dFC state transitions in the HCG. Taken together, these results provided empirical evidence for a linkage between the flexible reconfiguration of dynamic functional connectivity states and creative ability. These findings have the potential to provide new insights into the neural basis of creativity.

In the past decade, researchers have been trying to demystify how the creative brain generates novel and useful ideas. Research into the neural basis of creative cognition has increasingly focused on exploring large-scale brain functional networks, of which the default-mode networks (DMN) is one of the most widely studied networks^1^,^2^. The DMN comprises the medial prefrontal and medial temporal, inferior parietal, posterior cingulate, and lateral temporal cortices^3^ and is thought to be associated with several cognitive processes that have close ties to creativity, such as mind-wandering, mental simulation, and perspective taking^2^,^3^. The role of the DMN in creativity has been investigated in a number of neuroimaging studies. The precuneus (a core hub of the DMN), for example, has been reported in both structural^4^ and functional^5^,^6^ imaging studies of divergent thinking. In addition, other core hubs of the DMN, such as the anterior cingulate cortex^7^, posterior cingulate cortex and medial prefrontal cortex^8^, and the inferior parietal lobule^9^, are also associated with creativity. Additionally, previous studies also revealed the important role of the cognitive control network (CCN)^10^,^11^,^12^, which comprises the lateral prefrontal and anterior inferior parietal regions and is thought to be involved in planning, monitoring, and adapting behavior^13^, in creativity. The regions of the CCN have been found to engage in several creative tasks, including divergent thinking^10^, musical improvisation^11^, and artistic drawing^12^. Beaty and colleagues^2^ proposed a framework in which creative cognition arises from interaction between the DMN and CCN. Several studies also found evidence that creative cognition is a complex mental process which requires the integration of different neural circuits, such as the visual, auditory, and cerebellar networks^14^.

These studies have made substantial progress toward understanding the neural basis of creativity^2^,^14^. However, several fundamental issues still remain elusive. One central question is how the creative brain flexibly organizes different information in novel and unprecedented ways. The corresponding neural mechanism seems to be highly associated with a dynamic interplay between multiple brain regions. However, these studies, which quantified functional connectivity (FC) as the correlation between the time series of different regions across a whole scan without indicating the mediation of temporal covariation, are very limited in their ability to reveal this dynamic interplay. More recent, functional magnetic resonance imaging (fMRI) studies have been motivated by the idea that the brain is inherently a dynamic system^15^,^16^,^17^,^18^,^19^. Pursuing this line of thinking can provide new insights into the organizational principles of the creative brain.

A growing number of studies have indicated that FC shows noticeable variations over a range of seconds to minutes, even in the resting state without external stimuli^20^,^21^,^22^ (for reviews, see refs 15, 17). This dynamic functional connectivity (dFC) exhibits highly structured spatiotemporal patterns in which a set of metastable FC patterns, known as dFC states, reliably recur across time and subjects^23^. The dFC states have been investigated in a variety of studies, which have shown that the dFC states are associated with ongoing cognition^21^, consciousness level^20^, flexible behavior^16^,^24^, neuropsychiatric disorders^25^, and development^26^. Researchers have also proposed that neural metastability, which is a dynamic regime in which neural ensembles flexibly engage and disengage rapidly, has facilitative effects on memory, optimal information processing capabilities, and flexible behaviors^16^,^24^. Correspondingly, a computational simulation study revealed that individuals with low metastability underperform in associative memory, speed of information processing, and cognitive flexibility^27^. A recent new perspective suggests that the abundance of the brain’s dynamic repertoire facilitates novel cognition and behaviors, making it possible to adapt to external task demands rapidly and efficiently^28^. Given the points enumerated above, it is reasonable to assume that the dynamics of state transitions (transitioning from one dFC state to another) could reflect neural metastability at another level. That is, the dynamics of state transitions enable multiple brain regions to engage and disengage flexibly in coordination without being locked into fixed interaction patterns, resulting in the plentiful repertoire of brain reconfigurations that are required to facilitate creative task performance.

Creativity is thought to rely on dynamically reconfiguring brain regions that interplay in transient and complex connectivity patterns^29^. Creative individuals often achieve creative insights or problem solutions either through flexible switching among approaches, categories, and sets or by overcoming “functional fixedness”^30^,^31^. Therefore, we hypothesized that the intrinsic activity of creative brains might have a high transition frequency of dFC states to facilitate the cognitive flexibility, divergent thinking, unconscious mind wandering, and other mental activities that are necessary for creativity performance.

## Results

To test our hypothesis, we collected resting-state fMRI (R-fMRI) data from participants in a high-creative group (HCG, *n* = 22) and a low-creative group (LCG, *n* = 22), who were selected from 180 healthy volunteers after testing the volunteers using the figural version of the Torrance Tests of Creative Thinking (TTCT-Figural)^32^ and Raven’s Standard Progressive Matrices test^33^. The TTCT-Figural consists of three activities: picture construction based on a pear shaped circle, picture completion based on incomplete figures, and picture construction based on repeated parallel lines. The creativity scores were evaluated based on four measures: fluency (the number of figural images drawn by each subject), originality (the number of statistically novel figures), flexibility (the variety of categories of objects drawn by each subject), and elaboration (the number of details added on the pictures). As shown in Table 1, the creativity scores of the HCG (*M* = 65.4, *SD* = 4.09) were significantly higher than those of the LCG (*M* = 38.20, *SD* = 5.85), *t*(42) = 17.96, *p* < 0.01, but there were no significant differences between the two groups in age or intelligence.

A dynamic functional connectivity analysis approach^23^ was used to detect the characteristics of the dynamics of the functional connectivity of the HCG and LCG. We first identified 42 intrinsic connectivity networks by using group independent component analysis (ICA). Then the sliding window approach was used to estimate the dynamic functional connectivity (dFC) for each subject, and the *k*-means algorithm was employed to divide the dFC windows into separate patterns. We further explored the hub structure of each dFC pattern and the group differences in the properties of the dFC.

### Intrinsic connectivity networks

**Figure 1:**
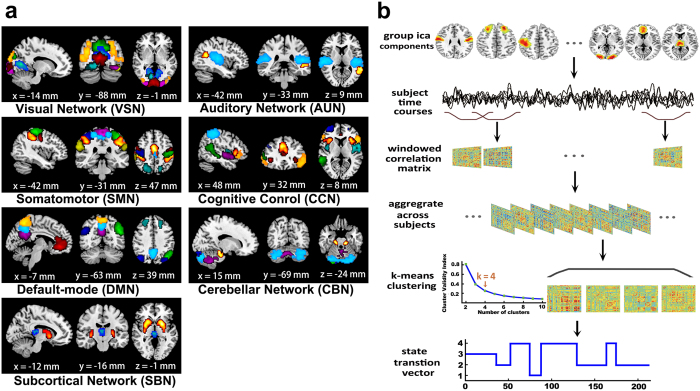
Intrinsic connectivity networks (ICN) identified from the resting-state fMRI (R-fMRI) data and the flowchart for the dynamic functional connectivity (dFC) analysis. (**a**) Identified ICNs, (**b**) flowchart for the dFC analysis. From this R-fMRI data, we used the sliding window approach to obtain 213 time windows and clustered the windows for all the participants using the k-means algorithm. The cluster centroids and cluster membership assignments that were obtained for all the windows represented the state transition vector.

A total of 42 intrinsic connectivity networks (ICNs) were identified using a group independent component analysis (ICA) approach (for details, see Supplementary Table S1). Based on their anatomical and functional properties, the ICNs were grouped into seven sub-networks: the auditory (AUN), cerebellar (CBN), cognitive control (CCN), somatomotor (SMN), default-mode DMN), visual (VSN), and subcortical (SCN) networks (Fig. 1a). This categorization of ICNs is consistent with that reported in a previous study^23^.

### Dynamic functional network connectivity

#### Connectivity patterns of dynamic functional connectivity states

**Figure 2:**
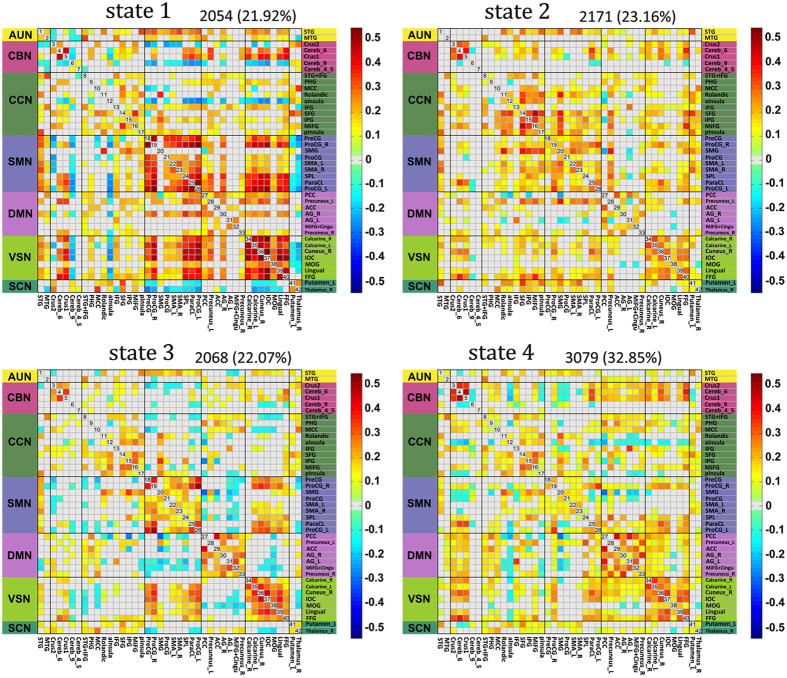
Connectivity patterns of the dynamic functional connectivity (dFC) states. The centroids of the dFC states are shown above. Only the significant connectivity for each state (FDR threshold (*q* < 0.05)) is shown. The total number and percentage of occurrences of each state are listed above each centroid. The full name of each region can be found in the supplementary material (list of abbreviations).

We used the sliding window approach^20^,^23^ to estimate the dFC network for each subject. Because the dFC patterns reoccur within subjects across time and across subjects^23^, we then applied the *k*-means algorithm^20^,^23^ to divide the dFC windows into four separate clusters. Figure 2 shows the centroids of the four dFC states. Briefly, state 1 showed a moderate to high correlation within and between the SMN and VSN. In state 2, the CCN and SMN displayed slight to moderate inter-network and intra-network connectivity, and the VSN showed moderate inter-network connectivity. The connectivity pattern of state 3 resembled that of state 1 but had a reduced strength in the SMN-, VSN- and CBN-related connectivity and an increased strength in the DMN. In state 4, the VSN displayed moderate inter-network connectivity; however, the DMN showed a moderate within-network connectivity.

#### Group differences and correlation with TTCT scores

**Figure 3:**
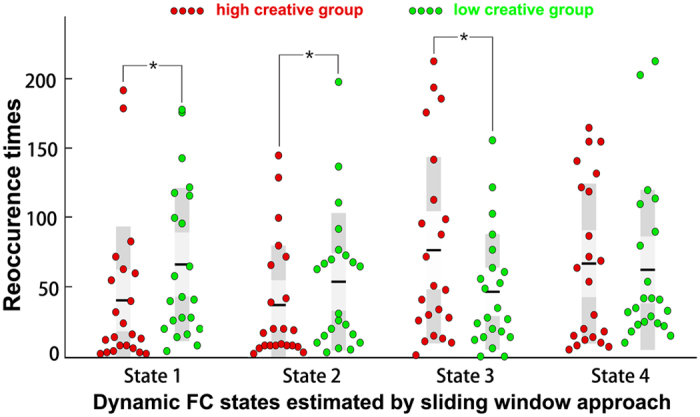
Group differences in reoccurrence times of the dynamic functional connectivity states. The reoccurrence times of each individual in high-creative and low-creative group are presented in red and green, respectively. For each group, the black line indicates the mean reoccurrence times of that group, and the light gray rectangle covers the data within one standard deviation above and below the mean. The pairs of groups with asterisks indicates that there are statistically significant differences between them.

**Figure 4:**
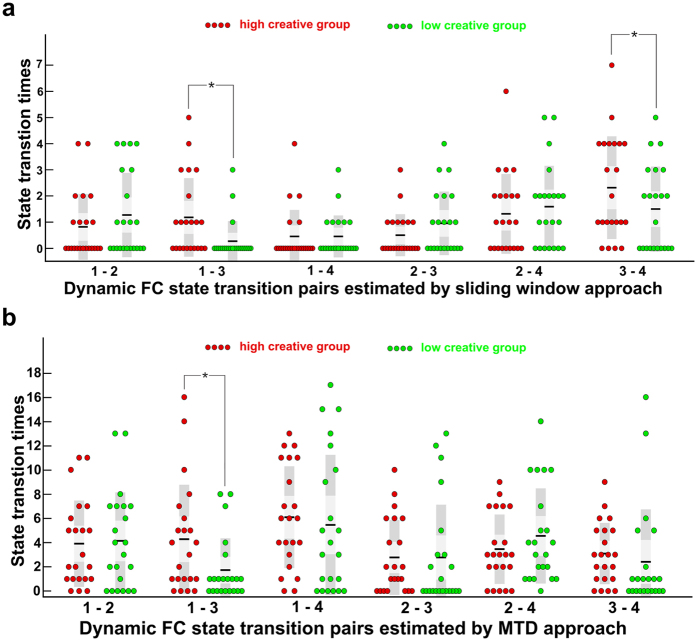
Group differences in the transition frequency of the dynamic functional connectivity states. (**a**) The dynamic FC states were estimated using sliding window approach; (**b**) The dynamic FC states were estimated using Multiplication of Temporal Derivatives (MTD). The ticks on the horizontal axis indicate the state transition pairs, e.g., “1–2” refers to the transitions between state 1 and state 2. The state transition times of each individual in high-creative and low-creative group are presented in red and green, respectively. For each group, the black line indicates the mean state transition times of that group, and the light gray rectangle covers the data within one standard deviation above and below the mean. The pairs of groups with asterisks indicates that there are statistically significant differences between them.

In this study, the properties of the dFC of each subject were depicted by two metrics, the reoccurrence times of all the dFC states and the dFC transition frequency between all the pairs of dFC states. For each subject, the reoccurrence time for each dFC state was defined as the total number of dFC windows assigned to that state, and the dFC transition frequency counts how many times the dFC windows altered their allegiance between a pair of states (e.g., the transition frequency between dFC state 1 and state 2 counted if the dFC windows altered their allegiance from state 1 to state 2 or from state 2 to state 1). Chi-square tests revealed significant differences in the reoccurrence times of the dFC states between the two groups (Fig. 3), *χ*^2^ (3, *N* = 44) = 423.01, *p* < 0.01. A post hoc analysis further indicated that the HCG had an increase in the reoccurrence times for state 3 but a decrease in the reoccurrence times for state 1 and state 2 compared to the reoccurrence times of the LCG (FDR corrected, *α* = 0.05). In addition, there were significant differences in the transition frequency of the dFC states between the two groups (Fig. 4a), *χ*^2^ (5, *N* = 44) = 14.97, *p* = 0.01. A post hoc analysis revealed that, compared with the LCG, the HCG had significantly more frequent transitions between state 1 and state 3 and between state 3 and state 4 (*p* < 0.05, uncorrected). To evaluate the robustness of our results, we performed exploratory analyses of *k* = 3 and 5 (please see supplementary information for further information). We obtained similar results, in that the high creativity group (HCG) had more frequent transitions between dynamic functional connectivity (dFC) states than did the low creativity group (LCG).

We further estimated the Pearson’s correlation of the individual TTCT scores with the reoccurrence times of the dFC states and the transition frequency of the dFC states for the HCG and LCG separately (Table 2). A correlation analysis revealed that the reoccurrence times of state 4 were positively correlated with the TTCT scores for the individuals in the HCG (*r* = 0.46, *p* = 0.03, uncorrected). Further, the frequency of the transition between state 3 and state 4 had a significantly positive correlation with the individual TTCT scores (*r* = 0.48, *p* = 0.03, uncorrected) in the HCG. For the LCG, there was no significant correlation with the individual TTCT scores or the reoccurrence times of the dynamic states (all *p > *0.1) or the frequency of the transition between them (all *p* > 0.2). In addition, there were no significant correlations between the transition frequency of the dFC state and the intelligence scores or age (all *p* > 0.05).

#### Validation using Multiplication of Temporal Derivatives

To improve the confidence of our results, we performed a confirmatory analysis using ‘Multiplication of Temporal Derivatives’ (MTD)^34^ to verify our hypothesis that whether the HCG would have more frequent transitions between the dFC states. The result of the MTD analysis revealed that the HCG had more frequent transitions between the dFC states than did the LCG. Chi-square tests revealed significant differences in the frequency of the transitions between the dFC states, *χ*^2^ (5, *N* = 44) = 26.70, *p* < 0.01. As shown in Fig. 4b, a post hoc analysis further indicated that the HCG had significantly more frequent transitions between state 1 and state 3 (FDR corrected, *α* = 0.05).

#### Hub structure of the dynamic functional connectivity states

**Figure 5:**
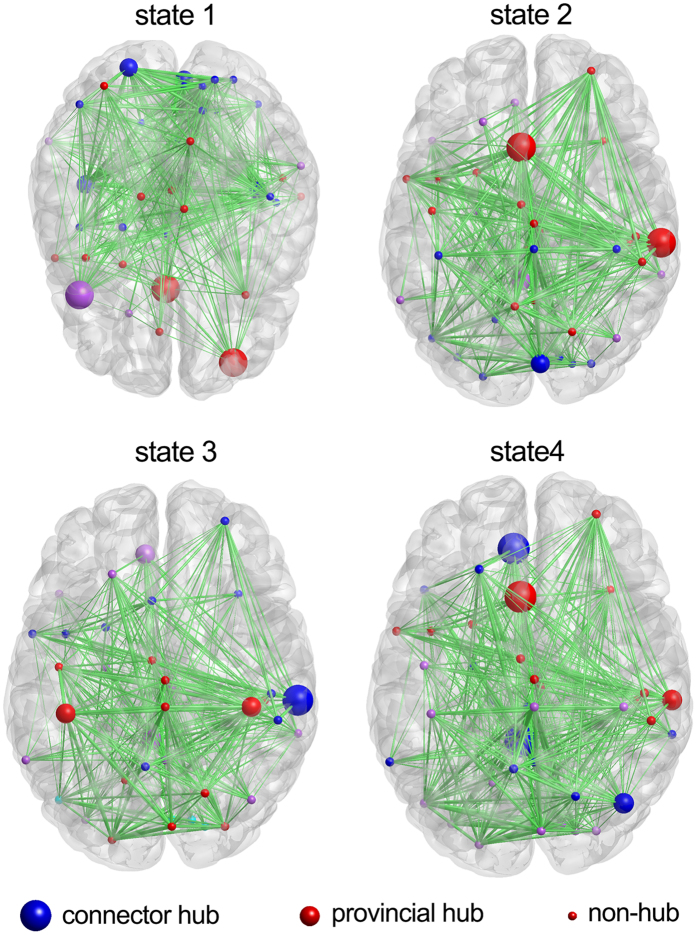
Hub structures of dynamic functional connectivity states. The nodes with the same color are assigned to the same module. The size of the nodes indicates their role: the largest are connector hubs, the smaller are provincial hubs, and the smallest are non-hub nodes.

To have a better understanding of the characteristics of the dFC states, we estimated the hub structures, which play a pivotal role in the organization of the overall networks, for each of the dFC states^35^. Figure 5 shows the hub structures of the four dFC states, and Table 3 lists all the modular hubs of each dFC state. Briefly, the hubs of state 1 were primarily located in the CCN, SMN and VSN, of these, three nodes in the CCN (inferior frontal gyrus, IFG; superior frontal gyrus, SFG; and middle frontal gyrus, MiFG) were connector hubs. The hubs of state 2 were scattered in the CCN, SMN, DMN and VSN, of these, the SFG in the CCN and the supra-marginal gyrus (SMG) in the SMN were connector hubs. The hubs of state 3 were located in the SMN and DMN, of these the SMG in the SMN was connector hub. Most of the hubs of state 4 were located in the DMN, and three nodes in the DMN (posterior cingulate cortex, PCC; anterior cingulate cortex, ACC; and angular gyrus, AG) and SFG in CCN were connector hubs.

## Discussion

The human brain is an inherently dynamic system^15^,^18^,^19^, and the flexible reconfiguration of brain networks is one of the remarkable characteristics of brain function which makes it possible to adapt to external task demands rapidly and efficiently, to generate novel ideas, and to flexibly switch between approaches, categories, and sets, or to overcome “functional fixedness”^28^. In the present study, we provided evidence that creative individuals have more flexible transitions between different dynamic functional connectivity states. These findings may provide new insights into the neural basis of creativity.

Researchers have developed a theoretical framework, metastability^16^, to describe these dynamics. The theory of metastability argues that the coordination of brain regions occurs flexibly with ensembles of a wide variety of sizes constantly coming together and disbanding in a repetitious cycle. This dynamic, self-assembly process achieves a dynamic equilibrium between the global integration of information and the local segregation of function^16^ and thus optimizes information transfer and processing capabilities^16^,^27^. The present study revealed that the HCG had a higher dFC state transition frequency between state 1 and state 3 and between state 3 and state 4 than that of the LCG (Fig. 4a). Further, the frequency of the transition between state 3 and state 4 was positively correlated with the creativity scores of the individuals in the HCG. A recent article pointed out that the abundance of the brain’s dynamic repertoire facilitates novel cognition and behaviors, making it possible to adapt to external task demands rapidly and efficiently^28^. The facilitative effects of the frequent transitions between the dFC states on creativity can be understood from the following perspectives.

First, a possible explanation of the present results comes from cognitive flexibility, which is one of the fundamental characteristics of creative individuals who are able to easily consider different perspectives and easily switch to different approaches. A strong link between cognitive flexibility and creative performance has been demonstrated in previous studies^7^,^36^. Recent studies have indicated that cognitive flexibility is highly associated with neural metastability^16^,^27^,^37^. A greater dFC state transition frequency enables the brain regions of creative individuals to engage and disengage more flexibly without experiencing locked interactions. The flexible switch from one connectivity pattern to another potentially explains the observations that creative individuals often achieve creative insights or problem solutions either through flexible switching between approaches, categories, and sets or by overcoming “functional fixedness”^30^,^31^.

Second, creativity might benefit from frequent transitions between the dFC states because of what is known as remote association. Previous studies have revealed that creativity benefits from the capacity to generate and use remote associations^38^. Less creative individuals generally strongly associate stimuli with only a few responses but do not strongly associate with other responses. Conversely, creative individuals are more likely to link seemingly irrelevant things to generate an unusual (original) response. Frequent transitions between dFC states with distinct connectivity patterns and hub distributions enables the brain to flexibly integrate information across multiple specialized sub-networks. Flexible information integration on such a large scale may increase the probability of linkage between seemingly irrelevant things.

Finally, frequent transitions between dFC states also enriches the repertoire of brain configurations, providing a foundation for novel ideas. Several researchers have argued that creativity involves a process that is similar to biological evolution: creative individuals are able to easily generate a large set of ideas, and although most of the new ideas will be useless and discarded as a result, the generated ideas may occasionally be both novel and appropriate^38^. The chance of generating a creative idea is small, but the chance increases when more ideas are generated. Frequent transitions between distinct connectivity patterns enables intensive information exchange and may have a facilitative effect on the generation of large sets of ideas.

In this study, we also found different dFC states that had distinct connectivity patterns and hub distributions of intrinsic brain activity. Additionally, the HCG had more reoccurrence times for dFC state 3 than did the LCG but had fewer reoccurrences for state 1 and state 2 (Fig. 3). Further, the reoccurrence times for dFC state 4 for individuals in the HCG were positively and significantly correlated with their TTCT scores. These results imply that the reoccurrence times for state 3 and state 4 are associated with high creativity. Compared with state 1 and state 2, state 3 and state 4 have more hubs in the DMN (Fig. 5 and Table 3). In particular, state 4 has three hubs in the DMN, and all three hubs are connector hubs that play important roles in inter-module connectivity and global information integration^35^. Many previous studies have argued that the DMN is highly associated with creativity through its contribution to the generation of candidate ideas or potentially valuable information^1^,^2^,^39^. In addition, a previous study^40^ found that the dynamic spontaneous activities of the DMN was associated with the degree of mind-wandering, which is tightly tied to creativity^41^. Thus, our results seem to provide further evidence that the DMN may play an important role in creative cognition.

There were several limitations that should be considered in future work. First, we measured creativity with the figural version of the TTCT, which primarily measures visual divergent thinking. However, we did not assess verbal divergent thinking; so whether verbal creative ability is related to similar or different patterns of brain network dynamics like that of visual creative ability remains elusive. In addition, although divergent thinking is a key element of creativity, divergent thinking only embodies one aspect of creativity; so a better understanding of creativity can only be obtained by using a more holistic perspective that covers divergent thinking as well as other mental process (e.g., convergent thinking). Second, the findings of the present study were based on data derived from normal healthy college students, but it should be determined whether the present findings also apply to other populations. The results of the present study should also be further validated by including individuals with proven high levels of creativity (i.e., inventors and artists). Third, the present study showed the association between dynamic networks of intrinsic brain activity and creativity. The interpretation of the dFC states is not straightforward, though many studies have indicated that it is associated with ongoing cognition^21^, consciousness level^20^, flexible behavior^16^,^24^, neuropsychiatric disorders^25^, and development^26^. The main finding of the present study that creativity ability was associated with the flexible organization of brain networks should be validated using other analysis methods, and the dynamic reconfiguration of brain networks while individuals are performing creativity-related tasks should also be investigated in further studies. Finally, our results should be interpreted cautiously. Since the dynamic functional connectivity analysis techniques in fMRI are relatively new and lack of a gold standard, the validity of our results needs to be verified by other analysis methods or further studies.

## Conclusions

The present study explored the association between the flexible reconfiguration of dynamic functional connectivity patterns and creativity ability. The findings showed that high creative individuals had a high frequency of transitions between dynamic functional connectivity patterns and that the transition frequency was positively correlated with creativity. These observations provided empirical evidence for the correlation between the flexibility of the reconfiguration of brain networks and creativity, findings which may help to elucidate the neural substrates underlying creativity.

## Materials and Methods

### Subjects

A total of 180 right-handed healthy volunteers (90 males, 18 ± 1.21 years) were recruited from South China Normal University (SCNU) in Guangzhou, China. None of the volunteers had a history of neuropsychiatric disorders or had previously received creativity- or art-related training according to their self-report. The experiment was conducted in accordance with protocols approved by the Review Board of SCNU. Written informed consent was obtained from each subject prior to the study.

The creativity performance of each subject was evaluated using the TTCT-Figural^32^. The TTCT is one of the most widely used measures of creativity and has a good construct validity^32^,^42^. The TTCT-Figural consists of three activities: picture construction based on a pear shaped circle, picture completion based on incomplete figures, and picture construction based on repeated parallel lines. In each of the activities, the subjects were asked to draw novel and meaningful pictures in ten minutes. For each subject, the creativity score was evaluated according to the TTCT scoring guidelines^43^. Briefly, the total scores for creativity were evaluated based on four measures: fluency (the number of figural images drawn by each subject), originality (the number of statistically novel figures), flexibility (the variety of categories of objects drawn by each subject), and elaboration (the number of details added to the pictures). Further details of the TTCT-Figural and its scoring details can be found in the Supplementary Information (SI). We also estimated the intelligence score for each subject using Raven’s Standard Progressive Matrices test^33^. Based on the TTCT scores, we selected 22 subjects (11 males) who were the top 12.22% of the population as the HCG and 22 participants (11 males) who were the bottom 12.22% as the LCG. In case this population of undergraduates had a relatively low diversity in their creative ability, we used a cut-off of 12.22%, rather than 25% or other more commonly used ratios, to magnify the difference in creativity ability between the two groups. The 12.22%, rather than the 10% or other cut-off point, was selected because we would get the same number of male and female subjects in both groups by using this cut-off point. Table 1 lists the ages, creativity scores, and intelligence scores for the 44 selected subjects. As shown in Table 1, the creativity scores of the HCG individuals were significantly higher than those of the LCG, but there are no significant differences between the two groups in age or intelligence. The 44 subjects were invited to participate in an MRI scan.

### Image acquisition

All the MRI data were acquired on a 3 T Siemens Trio Tim MR scanner with a 12-channel phased-array head coil at the Brain Imaging Center of SCNU. For each subject, the R-fMRI data were collected using a GE-EPI sequence with the following parameters: repetition time (TR) = 2,000 ms, echo time (TE) = 30 ms, flip angle = 90°, field of view (FOV) = 240 × 240 mm^2^, data matrix = 64 × 64, slice thickness = 3.5 mm, voxel size = 3.5 × 3.5 × 4.38 mm^3^, 32 transverse slices covering the whole brain, and 240 volumes. During the R-fMRI scan, each subject was requested to lie quietly with their eyes closed. In addition, we acquired high-resolution brain structural images by using a 3D multi-echo magnetization-prepared gradient echo (MP-RAGE) using the sequence: TR = 1,900 ms, TE = 2.52 ms, inversion time (TI) = 900 ms, flip angle = 9°, matrix size = 256 × 256, FOV = 256 × 256 mm^2^, slice thickness = 3.5 mm, voxel size = 1 × 1 × 1 mm^3^, 176 sagittal slices covering the whole brain.

### Data preprocessing

The R-fMRI data were preprocessed using SPM8 (http://www.fil.ion.ucl.ac.uk/spm). To account for magnetization equilibrium and the subjects’ adaptation to the experimental environment, the first 5 volumes were removed. The remaining images were corrected for the within-scan acquisition time delay between different slices, and then all the images were realigned to the first volume to correct for head motion. No subjects were excluded because of excessive head motion according to the criteria (above 1 mm displacement or 1° angular rotation). A two sample test indicated that there was no difference in the mean framewise displacement between the HCG (*M* = 0.13 mm, *SD* = 0.06) and the LCG (*M* = 0.11 mm, *SD* = 0.04), *t* (42) = 1.49, *p* = 0.14. After motion correction, the functional images were then co-registered to the T1-weighted structural images which were segmented into grey matter (GM), white matter (WM), and cerebrospinal fluid (CSF). The resulting GM images were nonlinearly registered with Montreal Neurological Institute (MNI) space, and the transfer parameters were then applied to normalize all the functional images. The normalized functional images were resampled to a resolution of 3 × 3 × 3 mm^3^ and smoothed with a Gaussian kernel of FWHM = 5 mm.

### Group independent component analysis

A group ICA was performed using GIFT (http://mialab.mrn.org/software/gift). In this study, we used a high model order group ICA (100 components)^44^ to segment the functional data of all the subjects. A principal component analysis (PCA) was used to reduce the data at two levels. First, the participant-specific data were reduced to 120 principal components (PCs). Then the reduced data of all the participants were further decomposed into 100 PCs using an expectation-maximization algorithm^45^. To estimate the reliability of the decomposition, we ran the Infomax group ICA 20 times in ICASSO. Back-reconstruction of the individual time courses (TCs) was performed and spatial maps (SMs) were made using the GICA1 algorithm. A total of 42 components were identified as intrinsic connectivity networks (ICNs). Finally, following a previous study^23^, we performed additional postprocessing steps on the time courses of the 42 ICNs, including (1) linear, quadratic, and cubic detrend; (2) low-pass filtering (0.15 Hz cutoff); (3) multiple regression of head motion; and (4) removal of detected outliers. It should be noted that we removed the outliers using an approach that is similar to the “scrubbing” method, but rather than deleting affected time points from the data, we replaced them with the best estimate, using a third-order spline fit to the clean portions of the time course^23^.

### Dynamic functional connectivity network

Figure 1b shows the flowchart of the dFC network analysis in this study. The main manipulations can be described as follows:

#### Sliding window approach

The sliding window approach^20^,^23^ was used to estimate the dFC network for each subject. We created tapered windows by convolving a rectangle (length = 22 TRs) with a Gaussian of *σ* = 3 TRs. The onset of each window progressively slid in steps of 1 TR from that of the previous one, resulting in 213 windows. The window length of 22 TRs was chosen because previous studies have shown that a window length of 22 TRs may provide a good trade-off between the quality of the functional network connectivity estimate and the temporal resolution^46^. In the calculations, we also imposed an L1 penalty on the precision matrix (inverse of the correlation matrix) to enhance scarcity^44^.

#### K-means clustering

Because the dFC patterns reoccur within the subjects across time and across the subjects, we applied the *k*-means algorithm to divide the dFC windows into separate clusters^20^,^23^. First, we estimated the variability of the dFC across all pairs at each window and selected the windows with local maxima in the FC variance as subject exemplars. Then, we performed a *k*-means analysis on the set of all the subject exemplars with a random initialization of the centroid positions. *k* = 4 was determined using the elbow criterion^25^. *k*s of 3 and 5 were each used to validate the robustness of our results (see SI for the details). We repeated the clustering algorithm 500 times to increase the chances of escaping the local minima. The correlation distance function was chosen because it is more sensitive to the dFC pattern, regardless of magnitude^23^. These resulting centroids were then used as starting points to cluster the dFC windows for all the subjects.

#### Hub structure of the dynamic functional connectivity states

The hub structure analysis was performed using the weighted and signed connectivity matrix for each dFC centroid. Before the analysis, each connection matrix for the cluster centroids was converted to a z-value connection matrix by using Fisher’s *r*-to-*z* transform to improve the normality. To reduce the risk of false-positive connections, we set the corrections to zero when their corresponding *p*-values were higher than a statistical threshold (FDR corrected, *α *=* *0.05). The hub structure was estimated by using the Brain Connectivity Toolbox (https://sites.google.com/site/bctnet/). Specifically, we first maximized the modularity using the Louvain algorithm with the resolution parameter *γ* = 1^47^. Given the stochastic nature of the Louvain algorithm, we repeated the estimation 100 times to get a consensus partition. Briefly, we computed the modular allegiance matrix *T* for each partition C_1_, C_2_… C_100_, whose binary elements *T*_ij_ imply whether node *i* was assigned to the same community with node *j*. The consensus matrix *T*_cons_ was obtained by summing all the modular allegiance matrices. We re-clustered the consensus matrix using the Louvain algorithm to get consensus communities^48^. We then calculated the module-degree *z*-score (within module strength) for each brain network^49^. According to the within-module degree, the nodes with *z* > 1 were identified as module hubs and nodes with *z* < 1 as non-hubs^50^. The hubs were then divided into “connector” and “provincial” hubs based on the level of their connectedness to other modules and their participation in local modules. The level of “inter-modular” connectivity compared to that of “intra-modular” connectivity of a node can be estimated by the participation index of a node^51^. Given a brain region *i*, the participation index is defined as


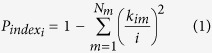


where *k*_*i*_ is the degree of node *i*; *N*_*m*_ is the number of modules; and *k*_*im*_ is the number of links from node *i* to module *m*. The hubs with a *P*index_*i*_ > 0.3 were defined as connector hubs (interconnecting module), and the hubs with a *P*index_*i*_ ≤ 0.3 as provincial hubs (connecting nodes within a module)^49^.

### Statistical analysis

Group differences in the reoccurrence times for each dFC state and the transition frequency for the dFC states were estimated using chi-square tests. The false discovery rate (FDR, *α* = 0.05) was used to correct for multiple comparisons. We further estimated the Pearson’s correlation between the TTCT scores and the reoccurrence times of the dFC states and between the TTCT scores and the transition frequencies of the dFC states within the HCG and LCG, separately. In the correlation analyses, the intelligence scores, age, and gender were used as covariates. To find out whether intelligence or age was related to the transition frequency of the dFC states, we also calculated the correlation between the transition frequencies and each of the variables.

### Validation using Multiplication of Temporal Derivatives

To improve the confidence in our results, we performed a confirmatory analysis using ‘Multiplication of Temporal Derivatives’ (MTD, https://github.com/macshine/coupling/)^34^. The MTD has been shown to be more sensitive than sliding window correlation methods in detecting dynamic alterations in connectivity structure and are less susceptible to known spurious sources, such as global mean signal fluctuations and head motion^34^,^52^. The MTD estimated similar changes over time. Specifically, a positive value implies that the time series couple in the same direction (either both increasing or both decreasing), but a negative value indicates anti-coupling (one increases while the other is decreases). The value of the MTD can be interpretable as a signed and weighted adjacency matrix for each temporal window. Further details of the MTD analysis can be found in the Supplementary Information. We calculated the spatial similarity of the adjacency matrix across all the time points and all the subjects by using spatial Pearson’s correlations. We further applied the *k*-means clustering algorithm (k = 4) to assign each time point of each subject to a cluster index. A statistical analysis was performed to help to validate whether the HCG had more frequent dFC states transitions.

## Additional Information

**How to cite this article:** Li, J. *et al*. High transition frequencies of dynamic functional connectivity states in the creative brain. *Sci. Rep.* **7**, 46072; doi: 10.1038/srep46072 (2017).

**Publisher's note:** Springer Nature remains neutral with regard to jurisdictional claims in published maps and institutional affiliations.

## Supplementary Material

Supplementary Information

## Figures and Tables

**Table 1:** Age, creativity and intelligence scores of participants in this study.

	HCG (*n* = 22)		LCG (*n* = 22)		*p*
	*M*	*SD*	*M*	*SD*	
Age	18.86	1.08	19.14	0.99	0.39
Creativity	65.54	4.09	38.20	5.85	2.53E-21
Intelligence	54.95	4.85	55.68	3.15	0.56

Note: HCG, high-creative group; LCG, low-creative group; the statistic was obtained using two-sample two-tail *t*-tests.

**Table 2:** Correlation of TTCT with reoccurrence times of dFC states and state transitions.

	LCG		HCG	
	*r*	*p*	*r*	*p*
Reoccurrence				
state1	0.17	0.47	−0.12	0.60
state2	0.29	0.20	−0.24	0.30
state3	−0.24	0.29	−0.18	0.43
state4	−0.25	0.28	0.48	0.03
State transition				
state1-state2	0.21	0.36	0.05	0.82
state1-state3	−0.17	0.46	−0.04	0.87
state1-state4	0.36	0.11	0.11	0.64
state2-state3	−0.30	0.18	−0.29	0.21
state2-state4	0.20	0.39	−0.03	0.90
state3-state4	−0.29	0.20	0.47	0.03

**Table 3:** Hubs of each dynamic functional connectivity state.

	Node	Network	*P*index	Role of hub
State 1	IFG	cognitive control network	0.45	connector
	SFG	cognitive control network	0.42	connector
	MiFG	cognitive control network	0.43	connector
	ProCG_R	somatomotor network	0.21	provincial
	ProCG_L	somatomotor network	0.21	provincial
	IOC	visual network	0.21	provincial
	Cuneus	visual network	0.13	provincial
State 2	SFG	cognitive control network	0.51	connector
	Cuneus	visual network	0.49	connector
	SMG	somatomotor network	0.35	connector
	PCC	default-mode network	0.19	provincial
State 3	SMG	somatomotor network	0.48	connector
	ACC	default-mode network	0.21	provincial
	PCC	default-mode network	0.18	provincial
	ProCG_L	somatomotor network	0.16	provincial
	ProCG_R	somatomotor network	0.13	provincial
State 4	SFG	cognitive control network	0.59	connector
	AG	default-mode network	0.58	connector
	ACC	default-mode network	0.53	connector
	PCC	default-mode network	0.51	connector
	SMG	somatomotor network	0.13	provincial

**Note:** The full name of each region can be found in Supplementary Information (list of abbreviations).
