# Supplementary material for: High transition frequencies of dynamic functional connectivity states in the creative brain
Source: Sci Rep. 2017 Apr 6;7:46072. doi: 10.1038/srep46072 (PMC5382673; doi:10.1038/srep46072)
Supplement: Supplementary Information [file srep46072-s1.pdf]

Supplementary Information for

# High transition frequencies of dynamic functional connectivity states in the creative brain

Junchao Li[1a], Delong Zhang[1a], Aiying Liang[2], Bishan Liang[3], Zengjian Wang[1], Yuxuan Cai[1], Mengxia Gao[1], Zhenni Gao[1], Song Chang[1], Bingqing Jiao[1], Ruiwang Huang[1*], Ming Liu[1*]

[1] Center for the Study of Applied Psychology, Key Laboratory of Mental Health and Cognitive Science of Guangdong Province, School of Psychology, South China Normal University, Guangzhou, China

[2] Guangdong Science Center, Guangzhou, China

[3] College of Education, Guangdong Polytechnic Normal University

[a] These authors contributed equally to this work.

* Correspondence to:

Ruiwang Huang, PhD
Center for the Study of Applied Psychology
Key Laboratory of Mental Health and Cognitive Science of Guangdong Province
School of Psychology, South China Normal University, Guangzhou 510631, China,
E-mail: ruiwang.huang@gmail.com (RH);

or

Ming Liu, PhD
Center for the Study of Applied Psychology,
Key Laboratory of Mental Health and Cognitive Science of Guangdong Province
School of Psychology, South China Normal University, Guangzhou 510631, China,
E-mail: lium@scnu.edu.cn (ML)

# Supplementary Information

## 1. Torrance Test of Creative Thinking (TTCT)

The creativity performance of each subject was measured using the figural version of Torrance Test of Creative Thinking (TTCT-Figural) (Torrance 1966). The TTCT-Figural measured fluency, originality, flexibility and elaboration, which were based on Guilford's divergent-thinking factors (Guilford, 1959; Torrance, 1996). The TTCT-Figural comprises three parts: picture construction (SI Fig. 1a), picture completion (SI Fig. 1b), and repeated figures of lines (SI Fig. 1c). In picture construction task, the subjects were asked to construct a creative picture that told a story based on a circle. In picture completion part, the subjects needed to complete 10 different lines to novel and interesting pictures. In repeated figures of lines part, subjects were asked to construct novel and meaningful pictures based on 10 pairs of parallel lines. Each part should be finished in ten minus.

**SI Fig. 1.** Samples of the figural version of Torrance Test of Creative Thinking

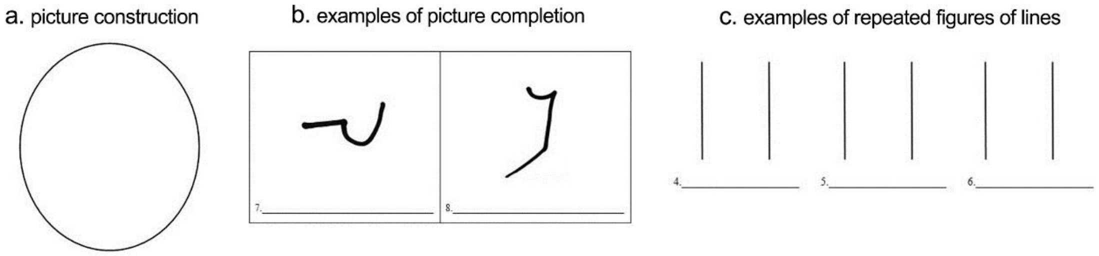


The creativity scores are determined based on four measures:

- *Fluency*: The number of relevant ideas; shows an ability to produce a number of figural images.
- *Originality*: The number of statistically infrequent ideas; shows an ability to produce uncommon or unique responses. The scoring procedure counts the most common responses as 0 and all
other legitimate responses as 1. The originality lists have been prepared for each item on the basis of normative data, which are readily memorized by scorers.
- *Elaboration*: The number of added ideas; demonstrates the subject's ability to develop and elaborate on ideas.
- *Flexibility*: scored by the variety of categories of relevant responses

**2. Number of dynamic FC states**

To validate the robust of our results, we performed exploratory analyses of k of 3 and 5. We obtained similar results that high creativity group (HCG) has more frequent transition among dynamic functional connectivity (dFC) states than low creativity group (LCG). Specifically, for k = 3, $\chi^2(2, N=44) = 8.44$, $p = 0.02$, post hoc analysis revealed that HCG had more frequent transition between dFC state 1 and state 2 than LCG; for k = 5, $\chi^2(9, N=44) = 24.59$, $p < 0.01$, post hoc analysis revealed that HCG had more frequent transition between dFC state 4 and state 5 than LCG.

**3. Validation using Multiplication of Temporal Derivatives**

To improve the confidence of our results, we performed a confirmatory analysis using 'Multiplication of Temporal Derivatives'. The MTD has been shown to be more sensitive than sliding window correlation methods in detecting dynamic alterations in connectivity structure and less susceptible to spurious connectivity, such as global mean signal fluctuations and head motion. The MTD estimated similar changes over time, specifically a positive value implies time series couple in the same direction (either both increasing or both decreasing), however negative value represents anti-coupling (one increasing while the other is decreasing). The value of the MTD can be interpretable as a signed and weighted adjacency matrix with each temporal window. To avoid the influence of high-frequency noise, we averaged MTD over a temporal window. Briefly, the MTD for the pairwise interaction between region *i* and *j* is defined according the following equation:

$$MTD_{ijt} = \frac{1}{w}\sum_{t}^{t+w}\frac{(dt_{it} \times dt_{ij})}{(\sigma_{dt_i} \times \sigma_{dt_j})} \qquad \text{(S1)}$$

Where $dt$ is the first temporal derivative of the $i$th or $j$th time series, $\sigma$ is the stand deviation od the temporal derivative time series for region $i$ or $j$, and $w$ is the window length of the simple moving average.

Given that we used a 0.15-Hz low-pass filter, all signals with periods of 15 seconds or shorter would be removed from the data in theory. Therefore, we used a temporal window with window length of 15 time points to calculate the moving average of the

MTD. The MTD can provide an estimation of time-varying functional connectivity. We calculated the spatial similarity of the adjacency matrix across all time points and subjects by using spatial Pearson's correlations. We further applied the K-means cluster algorithm (k = 4) to assigned each time point of each subject a cluster index. Statistical analysis was performed to validate whether the HCG has more frequent dFC states transition.

## 4. Abbreviation list

***Brain networks***

AUN: auditory network
CBN: cerebellar network
CCN: cognitive control network
SMN: somatomotor network
DMN: default-mode network
VSN: visual network
SCN: subcortical network
STG: superior temporal gyrus
MTG: middle temporal gyrus
Crus: cerebral crus
Cereb: cerebral
IFG: inferior frontal gyrus
PHG: hippocampus gyrus
MCC: middle cingulate cortex
PCC: posterior cingulate cortex
ACC: anterior cingulate cortex
AG: angular gyrus
aInsula: anterior insular cortex
pInsula: posterior insular cortex
MiFG: middle frontal gyrus
PreCG: precentral gyrus
ProCG: postcentral gyrus
SMG: supra-marginal gyrus
SMA: supplementary motor area
IOC: inferior occipital cortex
MOG: middle occipital cortex
FFG: fusiform gyrus
Lingual: lingual gyrus

***Other***

dFC: dynamic functional connectivity

TTCT: Torrance Tests of Creative Thinking

R-fMRI : resting-state fMRI

HCG: high creativity group

LCG: low creativity group

ICG: independent component analysis

FC: functional connectivity

fMRI: functional magnetic resonance imaging

PCA: principal component analysis

ICN: intrinsic connectivity

## 5. Supplementary table

**Supplementary Table 1. Peak Coordinates of ICNs**

| ICN regions | BA | tmax | Peak x | Peak y | Peak z |
|---|---|---|---|---|---|
| **Auditory networks** | | | | | |
| **STG(18)** | | | | | |
| R superior temporal gyrus | 41 | 22.26 | 48 | -27 | 115 |
| L superior temporal gyrus | 41 | 18.32 | -48 | -33 | 15 |
| **MTG(45)** | | | | | |
| L middle temporal gyrus | 21 | 15.99 | -60 | -54 | 18 |
| **Cerebellar networks** | | | | | |
| **Crus2(70)** | | | | | |
| L cerebelum crus2 | | 20.43 | -33 | -81 | -36 |
| **Cereb6(23)** | | | | | |
| R cerebelum 6 | 18 | 14.77 | 15 | -81 | -15 |
| **Crus1(24)** | | | | | |
| R cerebelum crus1 | 19 | 16.24 | 21 | -84 | -18 |
| **Cereb9(36)** | | | | | |
| R cerebelum 9 | | 21.03 | 9 | -54 | -39 |
| **Cereb4_5(7)** | | | | | |
| L cerebelum 4/5 | | 16.52 | -15 | -27 | -21 |
| **Cognitive control networks** | | | | | |
| **STG+IFG(21)** | | | | | |
| R middle temporal gyrus | 22 | 22.78 | 63 | -42 | 9 |
| R inferior frontal gyrus | 47 | 11.74 | 48 | 33 | -3 |
| **PHG(29)** | | | | | |
| L hippocampus gyrus | 35 | 18.56 | -24 | -12 | -15 |
| **MCC(21)** | | | | | |
| R middle cingulum gyrus | 23 | 23.81 | 0 | -30 | 30 |
| **Rolandic(55)** | | | | | |
| L rolandic oper | 48 | 16.37 | -57 | 3 | 6 |
| R rolandic oper | 48 | 12.38 | 60 | 3 | 6 |
| **aInsula(52)** | | | | | |
| L anterior insula | 47 | 19.91 | -30 | 24 | -3 |
| R anterior insula | 47 | 19.91 | 36 | 21 | -3 |
| **IFG(62)** | | | | | |
| L inferior frontal gyrus | 38 | 21.07 | -45 | 21 | -12 |
| R inferior frontal gyrus | 45 | 13.09 | 54 | 27 | 3 |

**(Supplementary Table 2 continued)**

| ICN regions | BA | tmax | Peak x | y | z |
|---|---|---|---|---|---|
| **SFG(78)** | | | | | |
| superior frontal gyrus | 24 | 21.03 | -3 | 18 | 39 |
| **IPG(96)** | | | | | |
| R inferior parietal gyrus | 40 | 22.68 | 54 | -36 | 51 |
| L inferior patietal gyrus | 40 | 17.19 | -51 | -42 | 54 |
| **MiFG(13)** | | | | | |
| R middle frontal gyrus | 46 | 18.77 | 30 | 54 | 24 |
| L middle frontal gyrus | 46 | 14.97 | -30 | 48 | 27 |
| **pInsula(11)** | | | | | |
| posterior insula | 13 | -42 | 3 | -3 | |
| **Somatomotor networks** | | | | | |
| **PreCG(1)** | | | | | |
| R precentral gyrus | 43 | 20.21 | 57 | -6 | 27 |
| L precentral gyrus | 44 | 21.47 | -45 | 12 | 33 |
| **R ProCG(4)** | | | | | |
| R postcentral gyrus | 3 | 19.85 | 42 | -30 | 54 |
| **SupraMariginal(65)** | | | | | |
| R supraMarginal | 48 | 18.38 | 63 | -27 | 23 |
| L supraMarginal | 40 | 13.68 | -63 | -33 | 30 |
| **PoCG(71)** | | | | | |
| R postcentral gyrus | 3 | 19.77 | 51 | -24 | 42 |
| L supraMarginal | 1 | 17.58 | -60 | -27 | 39 |
| L precentral gyrus | 6 | 12.12 | -54 | 6 | 30 |
| **L SMA(92)** | | | | | |
| L supplementary motor area | 6 | 21.59 | -3 | -9 | 51 |
| **R SMA(93)** | | | | | |
| R supplementary motor area | 6 | 20.9 | 3 | -18 | 57 |
| **SPL(47)** | | | | | |
| R superior parietal gyrus | 7 | 22.27 | 21 | -69 | 54 |
| L superior parietal gyrus | 7 | 15.01 | -18 | -69 | 48 |
| **ParaCL(27)** | | | | | |
| R paracentral lobule | 6 | 20.58 | 3 | -30 | 63 |
| **L ProCG(8)** | | | | | |
| L postcentral gyrus | 3 | 19 | -42 | -33 | 54 |

| ICN regions | BA | tmax | Peak x | Peak y | Peak z |
|---|---|---|---|---|---|
| **Default mode networks** | | | | | |
| **PCC(50)** | | | | | |
| L posterior cingulum | 26 | 27.94 | -3 | -45 | 27 |
| L medial frontal gyrus | 11 | 16.42 | -3 | 51 | -9 |
| **L Precuneus(51)** | | 23.77 | -6 | -57 | 57 |
| **ACC(20)** | | | | | |
| L anterior cingulum | 11 | 22.15 | -6 | 39 | -3 |
| **R AG(32)** | | | | | |
| R angular | 19 | 21.2 | 42 | -72 | 39 |
| L middle occipital gyrus | 19 | 19.73 | -42 | -78 | 33 |
| **L AG(40)** | | | | | |
| L angular | 39 | 23.63 | -48 | -66 | 30 |
| L middle cingulum | 23 | 11.78 | -3 | -42 | 39 |
| **MiFG+Cingulum(54)** | | | | | |
| R middle frontal gyrus | 8 | 17.53 | 27 | 27 | 48 |
| L middle frontal gyrus | 8 | 20.79 | -21 | 30 | 51 |
| R middle cingulum gyrus | 31 | 7.72 | 3 | -33 | 39 |
| **R Precuneus(95)** | | | | | |
| R precuneus | 7 | 23.81 | 3 | -54 | 42 |
| **Visual networks** | | | | | |
| **R Calcarine(10)** | | | | | |
| R calcarine | 17 | 24.09 | 6 | -81 | 6 |
| **L Calcarine(12)** | | | | | |
| L calcarine | 17 | 24.9 | -15 | -63 | 9 |
| **Cuneus(14)** | | | | | |
| R cuneus | 19 | 20.07 | 6 | -84 | 33 |
| **IOC(39)** | | | | | |
| L inferior occipital cortex | 18 | 16.5 | -21 | -90 | -9 |
| R inferior occipital cortex | 18 | 16.28 | 24 | -93 | -3 |
| **MOG(49)** | | | | | |
| L middle occipital gyrus | 18 | 17.25 | -24 | -93 | 12 |
| R middle occipital gyrus | 18 | 18.76 | 30 | -84 | 21 |
| **Lingual(68)** | | | | | |
| R lingual | 18 | 17.72 | 15 | -69 | -9 |
| **FFG(74)** | | | | | |
| R fusiform | 37 | 14 | 39 | -60 | -15 |
| L inferior occipital fyrus | 19 | 16.03 | -45 | -72 | -18 |
| **Subcortical networks** | | | | | |
| **Putamen(19)** | | | | | |
| L putamen | | 24.77 | -24 | 6 | 3 |
| **Thalamus(42)** | | | | | |
| R thalamus | | 20.58 | 6 | -21 | 3 |
